# Spatiotemporal Dynamics of Sleep Spindle Sources Across NREM Sleep Cycles

**DOI:** 10.3389/fnins.2019.00727

**Published:** 2019-07-10

**Authors:** Valentina Alfonsi, Aurora D’Atri, Maurizio Gorgoni, Serena Scarpelli, Anastasia Mangiaruga, Michele Ferrara, Luigi De Gennaro

**Affiliations:** ^1^Department of Psychology, Sapienza University of Rome, Rome, Italy; ^2^Department of Biotechnological and Applied Clinical Sciences, University of L’Aquila, L’Aquila, Italy; ^3^IRCCS Santa Lucia Foundation, Rome, Italy

**Keywords:** sleep spindles, slow/fast spindles, EEG source localization, LORETA, time course

## Abstract

The existence of two different types of sleep spindles (slow and fast) is well-established, according to their topographical distribution at scalp- and cortical-level. Our aim was to provide a systematic investigation focused on the temporal evolution of sleep spindle sources during non-rapid eye movement (NREM) sleep. Spindle activity was recorded and automatically detected in 20 healthy subjects. Low resolution brain electromagnetic tomography (LORETA) was applied for the EEG source localization. Aiming to evaluate the time course of the detected slow and fast spindle sources, we considered the first four NREM sleep cycles and divided each cycle into five intervals of equal duration. We confirmed the preferential localization in the frontal (Brodmann area 10) and parietal (Brodmann area 7) cortical regions, respectively for slow (11.0–12.5) and fast (13.0–14.5) spindles. Across subsequent NREM sleep episodes, the maximal source activation remained systematically located in Brodmann area 10 and Brodmann area 7, showing the topographical stability of the detected generators. However, a different time course was observed as a function of the type of spindles: a linear decrease across subsequent cycles was found for slow spindle but not for fast spindle source. The intra-cycle variations followed a “U” shaped curve for both spindle source, with a trough around third and fourth interval (middle part) and the highest values at the beginning and the end of the considered temporal window. We confirmed the involvement of the frontal and parietal brain regions in spindle generation, showing for the first time their changes within and between consecutive NREM sleep episodes. Our results point to a correspondence between the scalp-recorded electrical activity and the underlying source topography, supporting the notion that spindles are not uniform phenomena: complex region- and time-specific patterns are involved in their generation and manifestation.

## Introduction

Sleep spindles are transient oscillatory activity that appears in electroencephalography (EEG) during NREM sleep. They are typically defined as short (∼0.5–2 s) bursts of waxing and waning pattern within the sigma band (9–16 Hz) (De Gennaro and Ferrara, 2003).

The physiological function of these oscillatory patterns has not yet been completely elucidated. The evidence regarding their role in sleep maintenance appears controversial (Schabus et al., 2012; Sela et al., 2016), whereas it is well-known their critical role in learning and memory consolidation (Diekelmann and Born, 2010; Cox et al., 2014).

These phenomena are initiated in the reticular nucleus of the thalamus and the reciprocal interactions between specific regions of the cortex shape their duration and amplitude (Steriade and Llinás, 1988; McCormick and Bal, 1997; Ujma et al., 2019).

Spindles are traditionally classified into “slow” and “fast” subtypes, as a function of specific frequency and topography. The former is prevalent in the anterior cortical regions, while the latter spreads mainly in central and posterior areas (Werth et al., 1996; Zeitlhofer et al., 1997).

This regional distribution of scalp-recorded sleep spindles is also displayed at cortical level by studies exploring the activation of underlying cortical generators (Andrillon et al., 2011; Ktonas and Ventouras, 2014). Non-invasive estimation of cortical generators of sleep spindles has been provided by simultaneous EEG and magnetoencephalography recording, through modeling sources as electric current dipoles (Shih et al., 2000; Urakami, 2008). An alternative method is the low-resolution electromagnetic tomography (LORETA) (Pascual-Marqui et al., 2002). LORETA solves the inverse problem and received validation from several fMRI (Vitacco et al., 2002; Mulert et al., 2004) and intracranial recordings studies (Zumsteg et al., 2006). The solution space is restricted to cortical gray matter and modeled as a grid of volume elements (voxels) in the digitized structural template [Montreal Neurological Institute (MNI); Talairach and Tournoux, 1988]. It estimates the intracranial current source density (CSD) underlying global or selected electrical activity relying on a set of scalp-recorded potential differences, consistently with the assumption that contiguous neurons are simultaneously and synchronously active.

A pioneering study investigated the cortical electrical sources of a selected number of fast and slow spindles using LORETA (Anderer et al., 2001). The authors localized simultaneously active spindle generators clustered in frontal and parietal areas. Regions with the highest LORETA activity were mostly identified in the medial prefrontal gyrus and precuneus, respectively for slow and fast spindles. Subsequent studies applying such technique have confirmed the activation of this fronto-parietal network underlying the two types of spindles (Ventouras et al., 2010; Bersagliere et al., 2013; Del Felice et al., 2014) (Table 1).

**TABLE 1:** Source localization studies of sleep spindle.

	**Frequency (Hz)**	**Brodmann area (BA)**	**Lobe**
Anderer et al.,2001	<13 >13	BA 9,10 BA 7	Frontal Parietal
Ventouras et al.,2010	11–16	BA 17	Occipital
Bersagliere et al.,2013	12–16	BA 5,6,10,11	Frontal, parietal
Del Felice et al.,2013	10–12	BA 10,11,6,45,46	Frontal
	12–14	BA 6,10,11,20,21, 37,40,43,18	Frontal, temporal, parietal, occipital
Del Felice et al.,2014	10–12	BA 9, 10, 6, 21	Frontal, temporal
	12–14	BA 6,10,20,21,7,10	Frontal, temporal, parietal

Together with the regional distribution, also the temporal dynamics across sleep cycles differ between the two types of spindles. Density (number of spindle/minute) of both types of spindles linearly increases across consecutive NREM episodes (Werth et al., 1996), while EEG power values show a gradual decrease of the spectral power for slow spindles and a concomitant increase for fast spindles across consecutive sleep cycles (Tanaka et al., 1997). Consistently with this phenomenon, the frequency (period) of spindles increases over the course of a night’s sleep (Dijk et al., 1993; Wei et al., 1999). The spindle density also varies within each sleep cycle. They are more abundant at the beginning and at the end of NREM sleep episodes, typically following a U-shaped curve (Aeschbach et al., 1997; De Gennaro et al., 2000b; Himanen et al., 2002, 2003).

Surprisingly, no study systematically investigated the spatiotemporal dynamics of sleep spindles across the night by assessing the time course of the activation level of their sources. To fill this gap, we applied LORETA to track the temporal evolution of the sleep spindle sources between and within each NREM sleep cycle.

To sum up, the aims of the present study were (a) to confirm the known involvement of frontal and parietal sources in spindle generation and (b) to explore their time-varying activation across different sleep episodes.

## Materials and Methods

### Subjects

Twenty healthy subjects (12 males and 8 females; age range = 20–29, mean age = 23.8 ± 2.45 years) were selected as volunteers from a university student population. The requirements for inclusion were: regular sleep duration and schedule (habitual sleep time: 12:00 am–8:00 am ± 1 h), no daytime nap habits, no excessive daytime sleepiness and no other sleep, medical, neurological or psychiatric disorder, as assessed by a 1-week sleep log and by a clinical interview.

Participants were asked to keep constant their sleep/wake cycle during the week before the experimental night, and their compliance was controlled by daily sleep log and actigraphic recordings.

All subjects provided written informed consent. The study was approved by the Institutional Ethics Committee of the Department of Psychology of “Sapienza” University of Rome and was conducted in accordance with the Declaration of Helsinki.

### Procedure

Each subject participated in this study across two consecutive nights. The first night was considered of adaptation, while the following was the experimental night. Polysomnographic (PSG) recordings performed during the experimental night only was considered for the present analyses.

The sleep recordings were carried out in an electrically shielded, sound-proof and temperature-controlled room. Bedtime was scheduled at midnight and ended after 7.5 h of accumulated sleep, as visually checked online by expert sleep researchers.

### Polysomnographic Recordings

PSG was recorded using an Esaote Biomedica VEGA 24 polygraph. EEG signals were analogically filtered (high-pass filter at 0.50 Hz and antialiasing low-pass filter at 30 Hz [−30 dB/octave]). The 19 unipolar EEG derivations (Fp1, Fp2, F7, F8, F3, F4, Fz, C3, C4, Cz, P3, P4, Pz, T3, T4, T5, T6, O1, O2) were placed according to the international 10–20 system and recorded from scalp electrodes with linked mastoid electrodes as a reference. Bipolar horizontal electrooculogram (EOG) was recorded from electrodes placed approximately 1 cm from the medial and lateral canthi of the dominant eye. The bipolar submental electromyogram (EMG) was also recorded. The time constants were as follow: 0.03 s for EMGs and 1 s for EOGs. Impedance of these electrodes was maintained below 5 kOhm.

The whole-night sleep recordings (19 EEG channels, EOG, and EMG) were digitalized with a sampling rate of 128 Hz.

### Data Analysis

#### Sleep Stage Scoring

Sleep stages were visually scored (Cz derivation, EMG, and EOG) according to Rechtschaffen and Kales (1968). The sleep stages were scored for every 20 s epoch. Ocular and muscle artifacts were carefully offline excluded by visual inspection. The sleep measures are reported in Table 2.

**TABLE 2:** Polysomnographic measures.

**Variables**	**Mean (*SD*)**
Stage 1 (%)	6.76 (2.21)
Stage 2 (%)	59.34 (6.09)
SWS (%)	9.74 (6.44)
REM (%)	24.14 (4.31)
Awakenings (#)	27.85 (7.42)
Arousals (#)	35.1 (18.49)
TST (min)	451 (29.24)
TBT (min)	473.08 (40.03)
SEI % (TST/TBT)	93.23 (0.02)

*Means and standard deviations (SD) of the PSG variables.*

#### Spindle Detection

The automatic spindle detection from the whole night recordings was performed by means of a customized algorithm in MATLAB (The Mathworks, Inc., Natick, MA, United States), adapted from previous studies (Ferrarelli et al., 2007, 2010; Plante et al., 2013, 2015; Gorgoni et al., 2016; D’Atri et al., 2018).

The EEG data for all NREM epochs were band-pass filtered (Chebyshev Type II) between 11 and 15 Hz (−3 dB at 10 and 16 Hz). The detection of a spindle occurred when the mean signal amplitude of each channel exceeded an upper threshold set at six times the mean single channel amplitude. The sleep spindle characteristics are reported in Table 3.

**TABLE 3:** Global spindle characteristics.

	**Slow spindle (11.0–13.0 Hz)**			**Fast spindle (13.0–15.0 Hz)**		
	**NREM 2**	**NREM 3**	**NREM 4**	**NREM 2**	**NREM 3**	**NREM 4**
Amplitude (μV)	12.13 (1.29)	10.91 (1.50)	10.58 (1.59)	11.79 (1.27)	10.70 (1.63)	10.43 (1.98)
Frequency (Hz)	12.52 (0.08)	12.53 (0.11)	12.56 (0.22)	13.75 (0.17)	13.67 (0.19)	13.74 (0.32)
Duration (s)	1.14 (0.07)	1.06 (0.08)	1.00 (0.24)	0.99 (0.05)	0.84 (0.06)	0.88 (0.23)
Density (number/min)	1.06 (0.34)	0.64 (0.68)	0.31 (0.36)	1.65 (0.39)	0.77 (0.72)	0.38 (0.30)

*Means and standard deviations (SD) of detected spindle as a function of sleep cycle (NREM 2,3,4).*

#### Source Localization

In order to estimate the strength and distribution of the intracranial sources (CSD, μA/mm^2^) of EEG activity in the spindle range, we used the freeware eLORETA (exact Low-resolution electromagnetic tomography). We used the most recent version at the time of performing this study (v20170220). eLORETA was applied to estimate the three-dimensional distribution of the current density vector field of sleep spindles. The solution space, restricted to cortical gray matter, is represented by 6239 voxels, with a spatial resolution of 5 mm.

All EEG data containing the detected spindles on each scalp derivation were extracted and segmented for the source localization. The beginning and end of each epoch (total duration: 1 s) were defined as the 0.50 s immediately preceding and following the temporal midpoint of spindles. No repeated epochs were considered in the final analysis.

Before the CSD calculation, all electrodes were adapted to the international 10–20 system and registered to the 3D Montreal Neurological Institute’s MNI152 space (Talairach and Tournoux, 1988), producing a spatial transformation matrix for the inverse solution. Then, we determined the LORETA power in the frequency domain via the cross-spectral matrices from the EEG. Finally, CSD maps were extracted through the LORETA pseudoinverse transformation of the information at level of the scalp to the underlying current inside each voxel.

Current source density maps were averaged within- and then between-participants, yielding a grand average for the final group.

**FIGURE 1:**
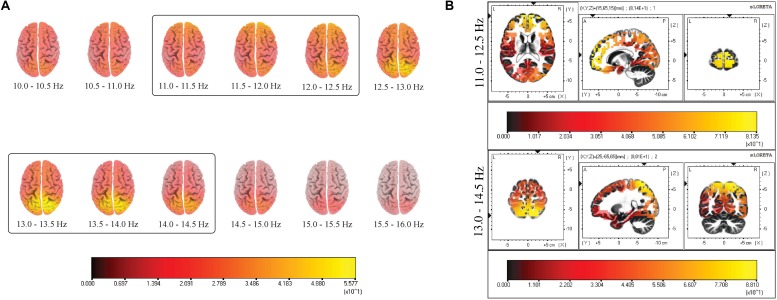
**(A)** Distribution of mean CSD of the whole spindle frequency range (10.0–16.0 Hz) with a frequency bin resolution of 0.5 Hz. The maximum of the estimated source is indicated in yellow. The prevalent source localization is situated over the anterior regions for low-frequency spindles and posterior regions for high-frequency spindles. **(B)** Distribution of mean CSD of slow (11.0–12.5 Hz) and fast (13.0–14.5 Hz) spindles in NREM sleep cycles. Two local maxima are identified: BA10 and BA7. Each distribution is shown relative to its maximum (in yellow), using axial, sagittal, and coronal slices intersecting at the point of maximal source.

A two-step process was performed to identify the active sources underlying slow and fast spindles. The first step consisted of estimating cortical sources for the whole spindle range under investigation (10.0–16.0 Hz) in the frequency domain, with a bin resolution of 0.5 Hz. In a second step, the three bins with the highest CSD were visually identified to define the respective range for slow (11.0–12.5 Hz) and fast (13.0–14.5 Hz) spindles, excluding the central bin due to the activation of both sources (Figure 1A). The resulting ROIs [in terms of Brodmann Areas (BA)] with maximal CSD values for slow and fast spindle range were considered as the putative source and used for subsequent analyses.

#### Time Course Analysis Between Cycles

With the aim of investigating the entity of possible variation across NREM episodes for the two identified spindle sources, we selected the first four consecutive NREM sleep episodes for each subject (i.e., the maximum common number of cycles).

Specifically, the NREM sleep episodes were operationally considered from the first epoch of NREM sleep to the onset of following REM sleep episode. No skipped first REM sleep episodes nor SO REM sleep episodes were present in our recordings.

We considered CSD as dependent variable, and it was computed separately for the slow and fast spindle sources. The CSD values of voxels within the ROIs were averaged.

Data were submitted to a two-way analysis of variance (ANOVA) with Spindle Type (Slow, Fast) and Sleep Cycle (1, 2, 3, 4) as within-subject factors. LSD method was used for planned comparisons (*p* ≤ 0.05).

#### Time Course Analysis Within Cycles

In order to assess the variations of cortical sources within each sleep cycle, we compared consecutive time frames. Due to the intra- and inter-individual variability in the duration of NREM sleep cycles, we divided each cycle into five intervals of equal duration, to make their time course comparable (Aeschbach and Borbély, 1993). For each interval of the cycles, CSD of slow and fast spindle sources was computed separately.

Aimed to assess time course within each sleep cycle, two-way ANOVAs considering Spindle Type (Slow, Fast) and Cycle Interval (1, 2, 3, 4, 5) as within-subject factors were carried out. LSD method was used for planned comparisons (*p* ≤ 0.05).

## Results

### Slow and Fast Spindle Sources

The results of source estimation of spindle activity from the 19-channel EEG recordings are depicted in Figure 1.

Figure 1B provides a grand average of CSD maps for slow and fast spindles during NREM sleep. LORETA processing revealed two broadly distinct cortical areas for slow and fast spindles, respectively placed in the anterior and posterior cortical brain areas. Multiple cortical sources for slow spindles were identified, scattered throughout the frontal areas. These areas included the superior and medial frontal gyrus (frontal lobe) and anterior cingulate (limbic lobe), with a maximum CSD value in the superior frontal gyrus (BA10). Multiple sources were also identified for fast spindles, mostly restricted and situated in the parietal areas (superior and inferior parietal lobule and post-central gyrus), with the superior parietal lobule (BA7) as the most active region.

The localization and MNI coordinates of local maxima CSD for each detected source of the two types of spindles are reported in Table 4.

**TABLE 4:** Local maxima of CSD distributions for slow (11.0–12.5 Hz) and fast (13.0–14.5 Hz) spindles.

	**Spindle 11.0–12.5 Hz**					**Spindle 13.0–14.5 Hz**				
**Local maximum**	**Lobe**	**Anatomical region (BA)**	**MNI coordinates**			**Lobe**	**Anatomical region (BA)**	**MNI coordinates**		
1	Frontal lobe	Superior frontal gyrus (10)	15	65	15	Parietal lobe	Superior parietal lobule (7)	25	−65	65
2	Frontal lobe	Superior frontal gyrus (10)	10	65	20	Parietal lobe	Superior parietal lobule (7)	20	−65	65
3	Frontal lobe	Medial frontal gyrus (10)	10	65	15	Parietal lobe	Post-central gyrus (7)	10	−60	70
4	Frontal lobe	Superior frontal gyrus (10)	20	65	10	Parietal lobe	Superior parietal lobule (7)	20	−60	65
5	Frontal lobe	Superior frontal gyrus (10)	5	65	15	Parietal lobe	Post-central gyrus (7)	15	−55	70

### Variations Between NREM Sleep Cycles

**FIGURE 2:**
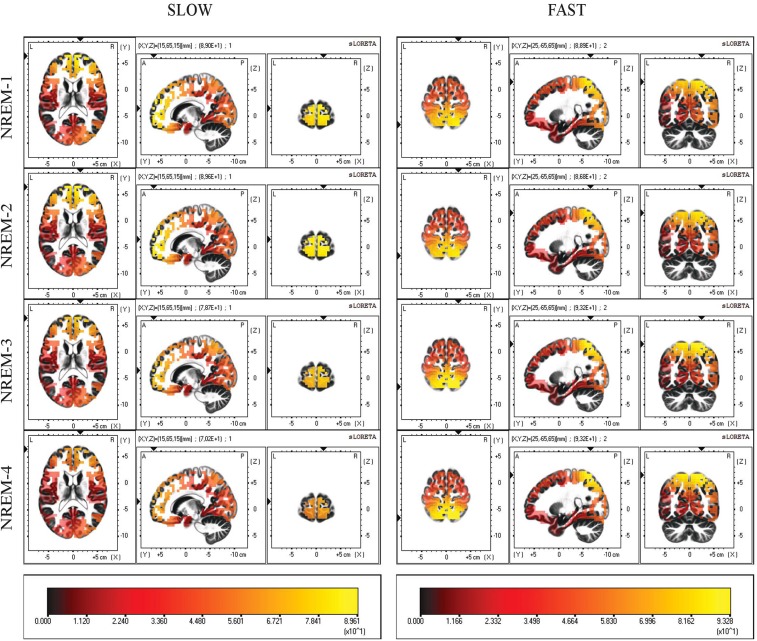
Distribution of mean CSD of slow (11.0–12.5 Hz) and fast (13.0–14.5 Hz) spindles in the first four NREM sleep cycle. Regardless of their current intensity values, local maxima of CSD remain in the same BAs (10,7) across all cycles. Each distribution is shown relative to its maximum (in yellow), using axial, sagittal, and coronal slices intersecting at the point of maximal source.

Current source density maps of group average in each NREM sleep episodes are depicted in Figure 2. Despite the varying intensity across cycles, we observed that the local maxima of CSD distribution for both slow and fast spindles remained stable.

**FIGURE 3:**
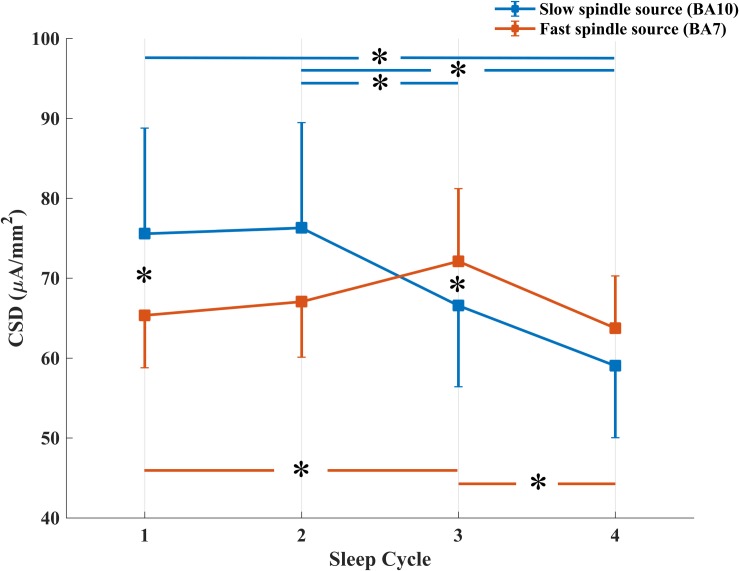
Temporal evolution of mean CSD in BA10 (blue line) and BA7 (orange line) across NREM sleep cycles. ^*^Signficant at *p* < 0.05.

The repeated measures ANOVA Spindle Type × Sleep Cycle on CSD values (Figure 3) showed a significant main effect of the Sleep Cycle (*F*_3,57_ = 3.279, *p* = 0.0273), in the direction of a clear reduction of CSD values in the fourth cycle compared to the first (*p* = 0.0223), second (*p* = 0.0099) and third (*p* = 0.0113) cycle. No significant main effect of Spindle Type was observed.

A significant interaction effect between the two factors was also observed (*F*_3,57_ = 4.264, *p* = 0.0087). LSD *post hoc* comparison showed that while CSD in BA10 (slow spindle source) progressively decreases over time (Cycle 1 vs. Cycle 4: *p* = 0.0012; Cycle 2 vs. Cycle 4: *p* = 0.0008; Cycle 2 vs. Cycle 3: *p* = 0.0504), CSD in BA7 (fast spindle source) showed a different trend. After an initial increase, statistically significant between Cycle 1 and Cycle 3 (*p* = 0.0266), we found a decrease from Cycle 3 to Cycle 4 (*p* = 0.0117). CSD of the two types of spindle was different in the first and third cycle, with a prevalence of BA10 within Cycle 1 (*p* = 0.0399) and of BA7 within Cycle 3 (*p* = 0.0477).

### Variations Within NREM Sleep Cycles

**FIGURE 4:**
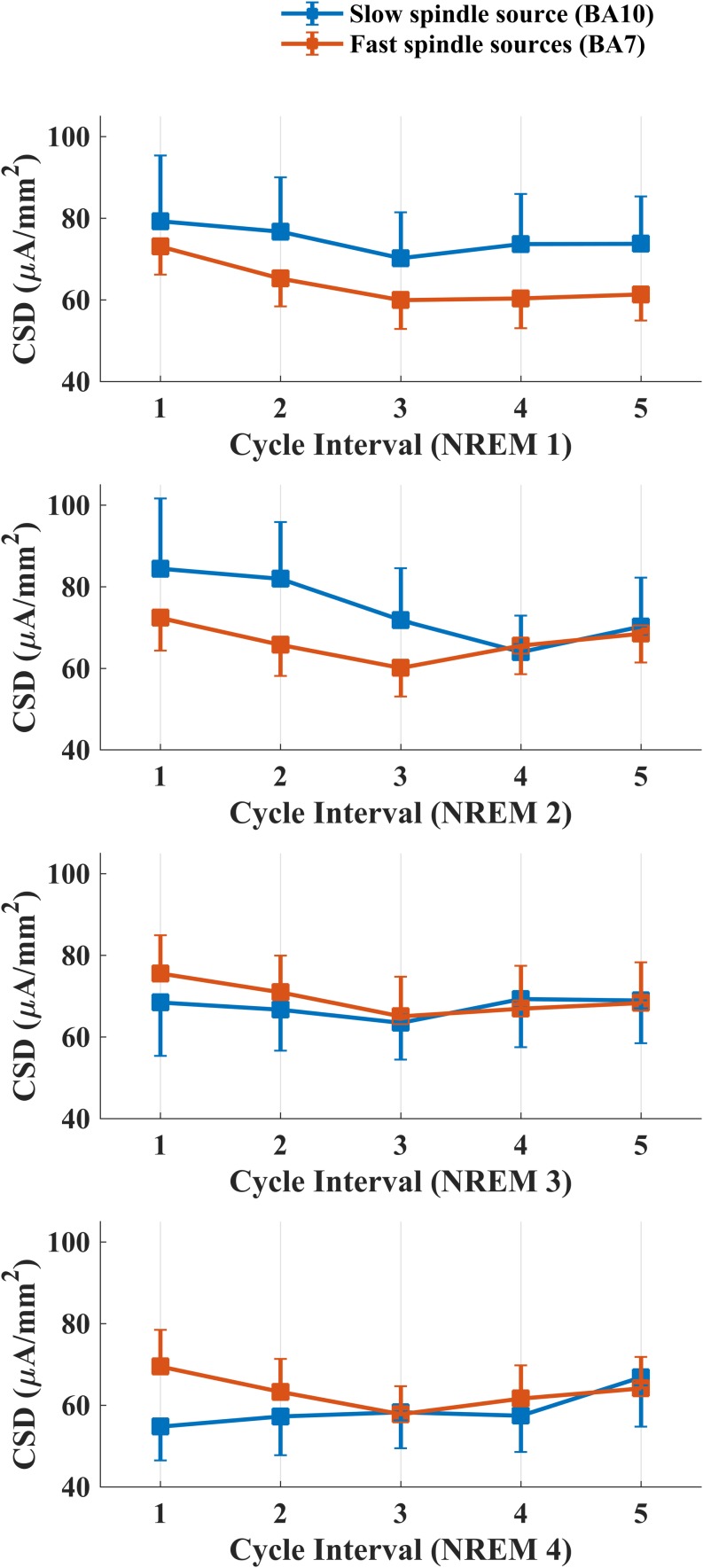
Temporal evolution of mean CSD in BA10 (blue line) and BA7 (orange line) across intervals within each NREM sleep cycle.

Figure 4 depicts intra-cycle variations of CSD computed from BA10 (slow spindle source) and BA7 (fast spindle source) during the first four sleep cycles.

We carried out four separate ANOVAs, one for each cycle, considering Spindle Type and Cycle Interval as within factors. A statistically significant main effect for the factor Cycle Interval was found for the first (*F*_4,76_ = 3.334, *p* = 0.0142) and second (*F*_4,76_ = 3.866, *p* = 0.0065) NREM cycle. No effect of Spindle Type or interaction Spindle Type × Cycle Interval was found in any NREM cycle.

LSD *post hoc* testing for the first NREM cycle showed a significant prevalence of CSD in the first interval as compared to the last three cycle intervals (Interval 1 vs. Interval 3: *p* = 0.0015; Interval 1 vs. Interval 4: *p* = 0.0083; Interval 1 vs. Interval 5: *p* = 0.01258). In the second cycle, we found the same decrease of CSD across different intervals (Interval 1 vs. Interval 3: *p* = 0.0031; Interval 1 vs. Interval 4: *p* = 0.0013; Interval 1 vs. Interval 5: *p* = 0.0296) and a prevalence of CSD in Interval 2 as compared to the Interval 4 (*p* = 0.0286).

## Discussion

To our knowledge, this is the first study specifically focused on temporal evolution of cortical spindle generators across the sleep night. Investigating the temporal dynamics of sleep spindle sources could potentially shed light on their involvement in spindle generation as a function of time.

According to the notion that spindling could reveal at least two functionally separated generators, respectively for high- and low- frequency spindles, we applied LORETA source estimation for two complementary purposes: (1) confirming the cortical areas which are involved in the generation of the two types of spindle and (2) tracking their changes between and within each NREM sleep cycle.

### Localization of Sleep Spindle Sources

We computed CSD maps in the frequency domain considering a wide frequency range (10.0–16.0 Hz) (Figure 1A), with a bin resolution of 0.5 Hz. This frequency-specific analysis identified the ranges 11.0–12.5 and 13.0–14.5 Hz as the frequency intervals with the highest values of CSD, respectively for the “slow” and “fast” spindle type. The frequency resolution allowed us to observe the smoothly changing of detected sources across consecutive bins, with a shift from the anterior activating areas (slower frequencies) to the posterior sources (faster frequencies).

The most active source of slow spindles was found in BA10, in the frontal cortex, while fast spindles range reached the absolute maximum in the parietal cortex (BA7) (Figure 1B).

The pattern observed at cortical level mirrors the appearance of spindles at the scalp level. This is consistent with the idea that EEG activity reflects the synchronous activation of brain electrical generators. Indeed, the slow spindle oscillations are predominant throughout the anterior derivations, while fast spindles prevail posteriorly (Werth et al., 1996; Zeitlhofer et al., 1997).

Despite the limited number of scalp electrodes (19) may have affected the precision of the inverse solution implemented by LORETA, the preferential localization of slow and fast spindles sources corresponds to those previously reported in high-density EEG studies (Del Felice et al., 2013, 2014). A strength of the current study is the use of a data-driven approach. Instead of *a priori* division of sleep spindles according to their topographical scalp distribution, the classification was only performed after the source reconstruction through LORETA. The aim was to avoid the pre-selection based on EEG activity.

### Time Course of Sleep Spindle Sources

To describe the temporal dynamics of such detected generators across the night, we first computed the average LORETA images for the first four NREM sleep cycles and then for the separate intervals within each cycle.

The maximum value of CSD remained systematically located in BA10 (slow spindle source) and BA7 (fast spindle source) along successive NREM sleep cycles, showing topographical stability of the detected generators (Figure 2). However, a gradual decline of CSD in BA10 over consecutive NREM episodes was observed. This decline was paralleled by a concomitant slight increase of CSD in BA7, reaching its peak around the third cycle and then decreasing again (Figure 3). The results provided by LORETA showed great inter-individual variability (see SEs in Figures 3, 4). Therefore, the possible absence of consistent pattern across subjects could make it difficult to generalize the observed findings.

Several evidences showed that while EEG power in slow spindle frequency range decreases over the night, an opposite trend is observed for higher frequency power (Tanaka et al., 1997; Cox et al., 2017). Similar results have also been reported for the amount (density) of slow and fast spindles across the night (Werth et al., 1996; Bódizs et al., 2009). Our results on active sources suggest an interesting resemblance with temporal dynamics exhibited by slow and fast spindles on scalp topography (spectral power, density).

The variations within cycles followed a “U” shaped curve for both spindle sources, with a trough around third and fourth interval (middle part) and the highest values at the beginning and the end of the time window investigated (Figure 4). As previously reported in EEG studies, such peculiar temporal pattern was also mirrored by EEG topography (Aeschbach et al., 1997; De Gennaro et al., 2000b; Himanen et al., 2002, 2003). The amount of sleep spindles and sigma activity power decreases with the deepening of sleep and increases closer to SO or REM transition (Silverstein and Levy, 1976; Guazzelli et al., 1986; Uchida et al., 1991, 1994; Purcell et al., 2017). In line with this evidence, numerous human and animal studies reported a negative temporal relationship between spindle activity and the low-frequency (0.5–4.0 Hz) and high-amplitude (≥75 μV) slow wave activity (SWA), which are predominant during SWS. Intracellular recordings suggest that spindle and delta waves (1.0–4.0 Hz) are generated by a common thalamocortical mechanism and their differentiation depends on the degree of membrane hyperpolarization of thalamocortical neurons (Steriade et al., 1993; Andrillon et al., 2011).

Moreover, also sleep pressure-related courses of SWA and spindle-frequency activity are negative correlated (Borbély et al., 1981; Dijk et al., 1993; De Gennaro et al., 2000a; Finelli et al., 2001). However, the response to sleep deprivation across the spindle frequency range is not uniform. The reduction of spindle activity only affects high-frequency spindle (>13.0 Hz) (Knoblauch et al., 2002), possibly reflecting a different sensitivity to homeostatic processes for the two types of spindles.

Therefore, the mutual relationship between these two EEG activities could explain both the variations of slow/fast spindle sources between NREM cycles (homeostatic decrease of slow spindle) and in the course of a NREM episode (reduced spindle activity in mid-cycle). However, the lack of comparison analysis between scalp- and source-recorded activities makes this interpretation speculative.

Hashemi et al. (2019) recently examined the interrelationship between sleep-spindles activity and higher frequencies (∼25.0 Hz). In their model, the cortical high frequency activity and its interaction with thalamic oscillation at fast spindle are proposed as key mechanism for the generation of slow spindles. Investigating the relationship between spindle sources and other patterns of brain activity could help understand how the local pattern of spindle activity (e.g., fast vs. slow) emerges. However, an exhaustive description of spindles local features is not possible with LORETA algorithm, due to its limited potential for the identification and localization of multiple and concomitant cortical sources (Nir et al., 2011; Piantoni et al., 2017; Hagler et al., 2018). Moreover, LORETA does not take into account co-localized sources, possibly explaining the key role of modulation of cortical spontaneous activity in the genesis of spindle (Hashemi et al., 2019).

Despite the observed time-related variations, the statistical comparison between slow and fast spindle sources was not significant. The two kinds of spindles, even if distinct, still showed strong overlapping time courses within each NREM episode. This can be interpreted in terms of similar cortical innervation patterns, regardless of different subcortical thalamic generators (Ujma et al., 2019). As highlighted in previous studies (Anderer et al., 2001; Bersagliere et al., 2013; Del Felice et al., 2014), the frontal and parietal spindle sources were directly connected with specific thalamic nuclei, where the spindles are initiated. Subsequent regional variability may stem from local neuronal activity, as suggested by previous researches with intracranial recordings (Andrillon et al., 2011; Nir et al., 2011; Ujma et al., 2019).

The debate about the existence of one or two sleep spindle generators should be considered open. The exploration of the spatiotemporal dynamics of cortical spindle sources could provide new information on this issue. However, given the key role of subcortical sources (thalamus, hippocampus) in spindle generation (McCormick and Bal, 1997; Sarasso et al., 2014), the LORETA solution space restricted to the cortex may represent an important limitation.

## Conclusion

Low resolution brain electromagnetic tomography combines the high time resolution of the EEG with a spatial accuracy in source localization, providing a reliable proxy about the complex region- and time-specific patterns of sleep spindles. Thanks to this reliable technique, we confirmed the involvement of the frontal and parietal brain regions in spindle generation. Moreover, we showed for the first time their changes within and between consecutive NREM sleep episodes. Our findings substantially confirm the available observations at the scalp level. Additionally, we can remark the concept that EEG activity reflects the synchronous activation of multiple sources at cortical level. In this regard, an interesting future direction would be the simultaneous exploration of both cortical sources and the corresponding EEG topography, to better characterize the dynamics and nature of the two types of spindles.

## Data Availability

The datasets generated for this study are available on request to the corresponding author.

## Ethics Statement

All subjects provided written informed consent. The study was approved by the Institutional Ethics Committee of the Department of Psychology of “Sapienza” University of Rome and was conducted in accordance with the Declaration of Helsinki.

## Author Contributions

VA, AD’A, and LDG conceived and designed the work. VA, AD’A, MG, SS, and AM acquired and analyzed the data. VA, AD’A, MG, and LDG interpreted the data. VA, AD’A, LDG, and MF drafted the work and revised it critically for important intellectual content. VA, AD’A, MG, SS, AM, MF, and LDG approved the final version of the manuscript and agreed to be accountable for all aspects of the work in ensuring that questions related to accuracy or integrity of any part of the work are appropriately investigated and resolved.

## Conflict of Interest Statement

The authors declare that the research was conducted in the absence of any commercial or financial relationships that could be construed as a potential conflict of interest.
